# Papillary Carcinoma Occurrence in a Thyroglossal Duct Cyst with Synchronous Papillary Thyroid Carcinoma without Cervical Lymph Node Metastasis: Two-Cases Report

**DOI:** 10.1155/2015/872054

**Published:** 2015-02-15

**Authors:** F. B. Sobri, M. Ramli, U. N. Sari, M. Umar, D. K. Mudrick

**Affiliations:** Surgical Oncology Division, Department of Surgery, Faculty of Medicine, Universitas Indonesia, Dr. Cipto Mangunkusumo General Hospital, Diponegoro Street 71, Jakarta 10430, Indonesia

## Abstract

*Background.* We present two rare cases of papillary carcinomas which appeared in thyroglossal duct cysts. These cases highlight that thyroglossal duct cyst can serve as malignancy of thyroid gland. *Methods.* A retrospective case report was carried out on 2 patients at Cipto Mangunkusumo Hospital. *Results.* A 57-year-old man presented with enlarged right anterior and midline neck mass, which preoperatively were diagnosed as thyroglossal duct cyst (TDC) and nontoxic multinodular goiter. A total thyroidectomy and Sistrunk procedure were performed. In the second case, a 35-year-old woman presented with a lump which occurred at anterior neck region without palpable mass at the thyroid. Preoperatively, it was diagnosed as TDC. Sistrunk procedure was performed, followed by total thyroidectomy a month after the first operation. Histopathology showed papillary thyroid carcinoma in both patients. *Conclusion.* The occurrence of carcinoma in TDC is very rare but should always be considered as an option in making diagnosis for a neck mass.

## 1. Introduction

The thyroid gland develops from the endodermal tissues of the primitive gastrointestinal tract. The site of origin ultimately is the foramen caecum at the tongue base and descent of the thyroid by the seventh gestational week to lie anterior to the cricoids and cervical trachea.

The descent of the thyroid through the anterior midline neck explains several anomalies that relate to thyroid pathology. Along the pathway of thyroid descent, a cyst of ciliated pseudostratified epithelium and variable amounts of thyroid tissue may remain which is called thyroglossal duct cysts [1].

The thyroid gland descends from the foramen cecum to the point below the thyroid cartilage by the seventh week of the intrauterine life. Thyroglossal duct is the epithelial connection between the thyroid gland and the foramen caecum. In the 8th to 10th week of gestation, this duct is normally obliterated. If a complete involution is failed, the remaining epithelium can develop a cystic expansion and form a thyroglossal duct cyst (TDC). TDC is the most common congenital anomaly in thyroid development and occurs in 7% of the adult population [2]. Thyroglossal duct carcinoma is very uncommon clinical pathology entity, occurring in approximately 1% of all the TDC [3].

## 2. Case Presentation 

### 2.1. Case 1

**Figure 1:**
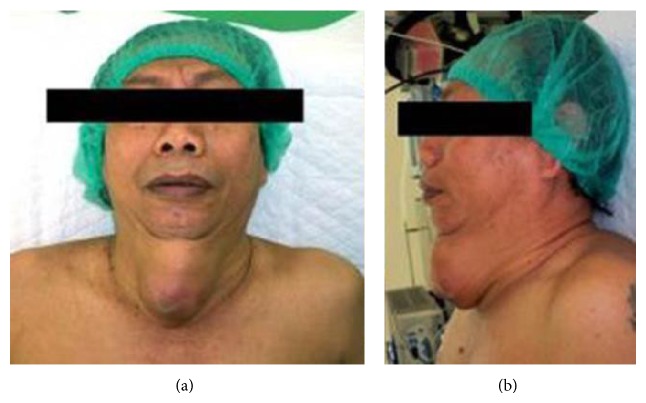
Clinical presentation of the patient.

A 57-year-old man presented to the oncology outpatient clinic with a year history of an enlarging right anterior neck mass and an enlarging midline neck mass since the last four months before admission. He was in a good health. On physical examination, there were a 5 × 3 × 2 cm firm mass on the right anterior part of the neck which is mobile with swallowing and a 6 × 5 × 5 cm soft tender cystic mass on the midline anterior (Figure 1).

A cervical ultrasound showed an irregular heterogenous echoic mass lesion which appeared to be a TDC and a left multinodular goiter.

**Figure 2:**
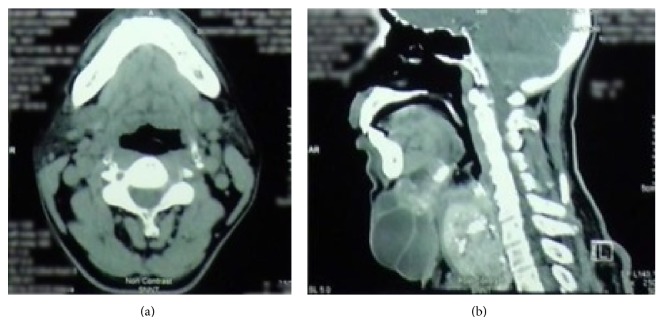
Axial and transverse section of a CT of the neck.

A cervical computed tomography (CT) showed a 6.24 × 4.53 × 6.57 cm heterogeneous calcified mass in the right anterior region of the neck extending to intrathoracic area and pushing the trachea to the left; a calcified left thyroid nodule measuring 0.73 cm in diameter; a 3 × 4 × 3.5 cm heterogenous mass destructing the hyoid bone which appeared to be malignancy; and a 7 × 9 × 5 cm septated cystic structure in the midline anterior of the neck (Figure 2).

At the presurgery stage, the lesions were diagnosed as a thyroglossal duct cyst (TDC) and a nontoxic multinodular goiter. Total thyroidectomy and Sistrunk procedure were performed to remove the tumor mass in TDC. The TDC tumor mass has already infiltrated the surrounding tissue so we performed debulking surgery and left very small remnant that was attached to the laryngeal mucosa.

**Figure 3:**
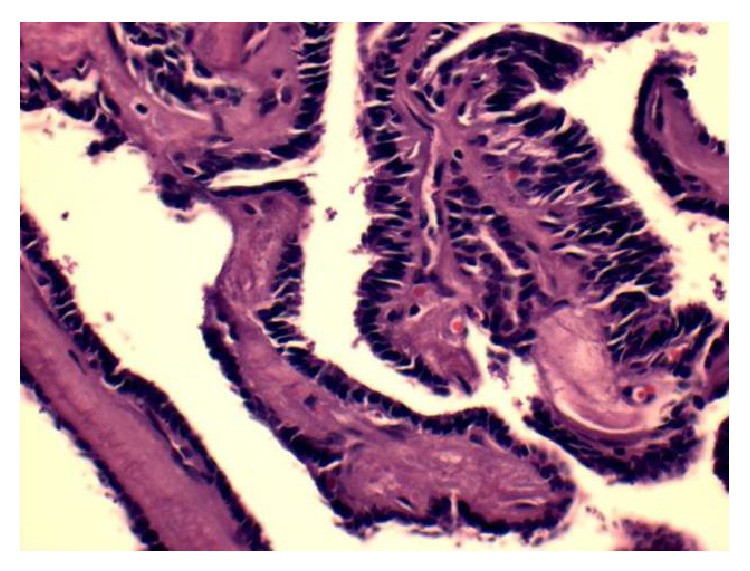
Papillary carcinoma “tall cell” variant evolving from a thyroglossal duct cyst (H&E).

At the postsurgery stage, the lesions were diagnosed as a papillary thyroid carcinoma “tall cell” variant arising in the TDC and both of the right and left (Figure 3). Pathological examination of the mass obtained from the second operation revealed papillary thyroid carcinoma.

The patient was treated with radioactive iodine ablation. The patient refused a follow-up visit after the radio iodine ablation; thus, we were unable to measure the thyroglobulin level in this patient.

### 2.2. Case 2

A 35-year-old woman presented with a lump at the anterior of her neck one month before admission, with no other complaints. Physical examination revealed a 3 × 3 × 3 firm mass with well-defined border which is mobile with swallowing. Laboratory investigation revealed no abnormalities.

At the presurgery stage, the lesions were diagnosed as a thyroglossal duct cyst (TDC) and the patient underwent a Sistrunk procedure to remove the lump.

At the postsurgery stage, the lesions were diagnosed as a papillary thyroid carcinoma. Thus, a total thyroidectomy was performed. A cervical ultrasound conducted before total thyroidectomy showed multiple small nodules in both lobes with calcifications and laboratory investigations were in normal range.

Postsurgery pathological examination of the mass obtained from the total thyroidectomy revealed papillary thyroid carcinoma. The patient refused a follow-up visit after the surgery; thus, the radioactive iodine ablation was not done. We were unable to measure the thyroglobulin level for this patient.

## 3. Discussion

A clinician should do a systematic approach when dealing with a neck mass since it is a common clinical finding that has extremely broad differential diagnosis. Most of the cases are due to benign process but clinician should be aware of malignant disease.

Thyroglossal duct is the epithelial connection between the thyroid gland and the foramen cecum. Normally, the duct will involute completely at the 8th to 10th week of gestation. The failure of this process causes the remaining epithelium to lead to the development of a thyroglossal duct remnants, most typically cysts [1]. TDCs are common congenital neck masses and were found in around 7% of adult population [2]. Although TDCs are usually benign, they may develop carcinoma in about 1% of cases and most of them arise from the ectopic thyroid tissue in the cyst. This entity [4] was first reported by Brentano (1911) and about 250 cases have been reported until today [5].

The etiology of the papillary carcinoma arising in a TDC is unclear but, generally, there are two theories which can explain this phenomenon, de novo origin and spread from a primary thyroid gland tumor [4]. Most authors support the theory of primary de novo origin by the ectopic thyroid nests of the cyst wall rather than the metastatic spread from a primary thyroid gland tumor through the duct from the thyroid carcinoma. The other theory explains synchronous occurrence of thyroglossal duct cyst carcinoma and thyroid carcinoma, which are reported to be even rarer, as multifocal tumor [6].

Strict criteria for TDC carcinoma diagnosis suggested by Mesolella et al. include a thyroglossal remnant, ectopic thyroid nests within the cyst wall, and a clinically normal thyroid gland [7]. Park et al. proposed criteria based on histopathological examination. A primary carcinoma of the thyroglossal duct should demonstrate that the duct or cyst has an epithelial lining with normal thyroid follicles within the cysts wall and normal thyroid tissue adjacent to the tumor and thyroid gland showing no sign of primary carcinoma [8].

The main difficulties faced in dealing with a carcinoma appearing in a TDC are the diagnosis and the management of this entity. Malignant TDCs are commonly mistaken from benign TDCs because of the indistinguishable mass presence at the anterior neck. Preoperative evaluation of thyroglossal duct cyst includes head and neck examination, palpation of thyroid gland, and imaging techniques. Malignancy cannot usually be diagnosed preoperatively by imaging diagnostic techniques (ultrasound, scintigraphy, and CT) [8]. Furthermore, FNAB (fine needle aspiration biopsy) can only give a correct result in about 53–66% of the cases [8, 9]. The diagnosis of carcinoma arising in TDC is based on pathological examination of the cyst. In most of the cases reviewed in the literature, the malignancy was not suspected before surgery but during surgery or from definitive pathological samples [10].

Beside the difficulties encountered in the diagnosis, clinicians also faced much of controversy regarding the surgical treatment arising in a TDC. Because TDC carcinoma is a rare case and lacks preoperative diagnosis, in addition to the possibility of associated thyroid malignancy, there is lack of consensus in the literature regarding the optimal management for TDC carcinoma [11].

Another difficulty we faced in Indonesia is a financial problem. Although public health insurance in Indonesia covers most of medical procedures, patients are still unable to afford another expenditure, such as transportation cost from their home to the hospital. This financial problem became the reason why the thyroglobulin level was not measured for both patients and the radioactive iodine ablation was not done for the second patient.

The surgical procedure performed commonly for a thyroglossal duct cyst is a surgery called Sistrunk's procedure. This procedure consists of removal of the thyroglossal duct cyst, the medial segment of the hyoid bone, and a core of tissue around the duct to open into the oral cavity at the foramen cecum. Although some surgeons consider the Sistrunk's procedure to be adequate if histological examination does not show extracystic extension [11], other literatures suggest more aggressive surgical approaches based on the finding that papillary thyroid carcinoma may spread through the thyroglossal duct remnant even with no lesion detected clinically in the gland itself. Those literatures suggest that patients be treated with Sistrunk procedure, total thyroidectomy, postoperative radioactive iodine therapy, and thyroid hormone replacement [7].

When the definitive histological analysis reveals malignancy after Sistrunk procedure to remove TDC, the thyroid gland must be studied further with radiological and scintigraphic examinations [12]. Clinicians are in dilemma to perform further managements including extension of surgery (such as total thyroidectomy) according to the criteria established for differentiated thyroid cancer, regional lymph node dissection, radioactive iodine, and suppressive thyroxine therapy. The reason for those suggested managements is the fact that incidences of primary thyroid carcinoma concomitant with TDC carcinoma are about 11–45% [2, 4], where total thyroidectomy was performed to those cases. Our case was not similar to those incidences because the thyroid carcinoma is found as bulk of tumor without much normal thyroid tissue left.

A total or subtotal thyroidectomy has been recommended if there is cystic wall invasion by the carcinoma or if the TDC carcinoma is greater than 1.5 cm [6]. Thyroid suppression is recommended for all patients regardless of the thyroid management [13]. We performed a total thyroidectomy for a very big thyroid carcinoma and the patient has received thyroid suppression therapy.

A previous study reported that papillary carcinoma in TDC has the same similarities with general papillary carcinoma, including the similarity in terms of lymph node metastasis [14]. Thus, neck dissection is performed only when lymph node metastases are found on ultrasound or during surgery. The type of neck dissection is limited to central compartment neck dissection in keeping with these midline tumours. In these two patients, we did not find lymph node neck metastasis. Therefore, as a guideline recommended by most of the oncology centers around the world, lateral neck dissection was not done [14–17].

The prognosis for papillary TDC carcinoma is excellent, with occurrence of metastatic lesions in less than 2% of cases [2]. In this case, histopathology examination showed papillary thyroid carcinoma “tall cell” variant within the TDC and almost all of thyroid area. A recent cohort study comparing “tall cell” variant patients with usual variant papillary thyroid cancer patients revealed that 5-year disease specific survival was poorer in the “tall cell” variant group. Along with it, the deaths number in the “tall cell” variant group was higher [18]. A meta-analysis study revealed that “tall cell” variant patients have higher rate of recurrence and mortality [19].

## 4. Conclusion

Formation of thyroid carcinoma in a TDC is very rare. Surgeons must be aware and include this entity while examining a patient with a neck mass especially located around hyoid bone [2] with physical examination suspected malignancy. Considering the embryological development of the thyroid, the ideal treatment for the malignancy of the thyroglossal duct consists of total thyroidectomy too.
